# The Brazilian Rare Genomes Project: Validation of Whole Genome Sequencing for Rare Diseases Diagnosis

**DOI:** 10.3389/fmolb.2022.821582

**Published:** 2022-05-02

**Authors:** Antonio Victor Campos Coelho, Bruna Mascaro-Cordeiro, Danielle Ribeiro Lucon, Maria Soares Nóbrega, Rodrigo de Souza Reis, Rodrigo Bertollo de Alexandre, Livia Maria Silva Moura, Gustavo Santos de Oliveira, Rafael Lucas Muniz Guedes, Marcel Pinheiro Caraciolo, Nuria Bengala Zurro, Murilo Castro Cervato, João Bosco Oliveira

**Affiliations:** Hospital Israelita Albert Einstein, São Paulo, Brazil

**Keywords:** rare diseases, precision medicine, genomics, genetic diagnostic test, whole genome sequencing

## Abstract

Rare diseases affect up to 13.2 million individuals in Brazil. The Brazilian Rare Genomes Project is envisioned to further the implementation of genomic medicine into the Brazilian public healthcare system. Here we report the validation results of a whole genome sequencing (WGS) procedure for implementation in clinical laboratories. In addition, we report data quality for the first 1,200 real-world patients sequenced. We sequenced a well-characterized group of 76 samples, including seven gold standard genomes, using a PCR-free WGS protocol on Illumina Novaseq 6,000 equipment. We compared the observed variant calls with their expected calls, observing good concordance for single nucleotide variants (SNVs; mean F-measure = 99.82%) and indels (mean F-measure = 99.57%). Copy number variants and structural variants events detection performances were as expected (F-measures 96.6% and 90.3%, respectively). Our WGS protocol presented excellent intra-assay reproducibility (coefficients of variation ranging between 0.03% and 0.20%) and inter-assay reproducibility (coefficients of variation ranging between 0.02% and 0.09%). Limitations of the WGS protocol include the inability to confidently detect variants such as uniparental disomy, balanced translocations, repeat expansion variants, and low-level mosaicism. In summary, the observed performance of the WGS protocol was in accordance with that seen in the best centers worldwide. The Rare Genomes Project is an important initiative to bring pivotal improvements to the quality of life of the affected individuals.

## Introduction

Rare diseases represent a group of over 9,000 disorders affecting an estimated 114 to 470 million patients globally (1.5%–6.2% of the global population) (Ferreira, 2019). Rare diseases with genetic etiology are the leading cause of death in children, and the diagnosis is challenging (Lionel et al., 2018). Early genetic testing leads to clear benefits by reducing the time until diagnosis, leading to a better choice of therapeutic interventions, improving couples’ confidence in having children again, and reducing healthcare costs (Lionel et al., 2018).

The human genome was first mapped through the Human Genome Project (HGP), an extensive international collaboration over 13 years (Lander et al., 2001; Venter et al., 2001). Essential advances in sequencing technology, such as the development of next-generation sequencing (NGS), have enabled the sequencing of a complete genome within hours, at a fraction of the initial cost, which resulted in the generation of a large amount of data and a widespread application for diagnosis and research (Wetterstrand, 2020).

NGS encompasses several approaches: whole genome (WGS), whole exome (WES), and targeted (panel) sequencing. With WGS, it is possible to read approximately all three billion base pairs of the human genome (Nagarajan and Pop, 2013). The falling cost, increasing ease of application, and comprehensive nature of WGS make it the ideal tool for routine use in rare disease diagnosis.

WGS can overcome many of the technical limitations of other NGS approaches, including uneven coverage and low sensitivity for the detection of copy, number structural, and expansion repeat variants (Belkadi et al., 2015). In addition, it enables the identification of noncoding and mitochondrial variants (Bick et al., 2019). In fact, many studies have shown that WGS has a high diagnostic yield and that early molecular diagnosis improves outcomes and reduces healthcare costs (Vissers et al., 2017; Howell et al., 2018).

The WGS workflow can be divided into three major steps: wet laboratory sample processing, bioinformatics analyses for variant calling and annotation, and correlation of the clinical and molecular findings, resulting in a medical report. The implementation of WGS in clinical laboratories thus requires critical assay design, validation, and implementation of quality control measures according to specific guidelines recommendations to ensure adequate performance before use in diagnostic routine (Barra et al., 2018; Marshall et al., 2020).

The Brazilian Rare Genomes Project envisions further the implementation of genomic medicine into the Brazilian national public healthcare system (SUS), complementing current policies and significantly improving the diagnostic capacity for rare disorders. Moreover, as Brazilian populations have high genetic diversity and are underrepresented in ancestry and human genetic variation databases such as 1,000 Genomes (The 1000 Genomes Project Consortium, 2015), a secondary objective of the Brazilian Rare Genomes Project is to assess the ancestry of the participants, improving precision medicine in the country.

Here we report the results of the development and validation of a PCR-free WGS protocol for clinical use in the project, including wet-lab workflow and bioinformatics pipelines. In addition, we document the protocol performance in the first 1,200 samples sequenced.

## Material and Methods

### Sample Selection and Test Scope

Our validation dataset was composed of 76 samples (Supplementary Table S1). Among them, 22 were international reference samples purchased from Coriell Life Sciences (Philadelphia, PA, United States) for benchmarking and validation, including seven reference samples from Genome in a Bottle Consortium (GiaB) (12). The remaining 54 are samples previously characterized by other methodologies: conventional Sanger sequencing, single nucleotide polymorphism (SNP) array, array comparative genomic hybridization (aCGH), conventional karyotyping, or fluorescence *in-situ* hybridization (FISH). We intended to detect and report single nucleotide variants–SNVs, insertion/deletions–indels, copy number variants–CNVs (large deletions and duplications, chromosomal aneuploidy), and structural variants–SVs (inversions, translocations), as well as mitochondrial SNVs. Repeat expansions and mosaicism were not included in the scope of this first phase of validation.

Samples were sequenced across three independent workflows (library preparation → sequencing → data analysis). We selected two benchmark samples to assess reproducibility: the reference sample NA24385 was replicated into one workflow for intra-assay reproducibility evaluation, whereas NA24694 was included in all three workflows for inter-assay reproducibility evaluation. Different operators independently performed the workflows.

All methodological procedures were performed in the Hospital Israelita Albert Einstein CLIA/CAP-accredited laboratories (Aziz et al., 2015).

### Research Ethics Statement

This study adhered to the Declaration of Helsinki principles for research in human beings and was approved by the Hospital Israelita Albert Einstein’s Research Ethics Committee (São Paulo, Brazil. Protocol number: CAAE 29567220.4.1001.0071). All individuals provided written consent for WGS testing and use in research, since the Brazilian Rare Genomes Project will make variant and summary-level data available for public use through periodic submissions to databases such as ClinVar and Matchmaker Exchange.

### DNA Extraction, Quantification, and Fragmentation

DNA was extracted from whole blood samples using QIAsymphony DNA Mini Kit at QIAsymphony automated system (both Qiagen, Valencia, CA, United States). The extracted DNA was eluted into a final volume of 90 µl with an elution buffer. Genomic DNA purity was evaluated using NanoDrop 2000 (thresholds: 260/280 ratio ≈1.8 and 260/230 ratio between 1.8 and 2.2). DNA quantification was performed with Qubit^®^ 4 fluorometer using the Qubit^®^ dsDNA HS assay (both Life Technologies, Carlsbad, CA, United States). If sample input does not reach the desired quality, we reject it and request sample recollection.

Genomic DNA was fragmented into 350 bp inserts by Covaris ME220 ultrasonicator (Covaris, Woburn, MA, United States), with the following treatment settings–DNA input: 1 µg (final volume of 55 μl, completed with resuspension buffer); peak incident power: 50 W, duty factor: 20%, cycles per burst: 200, duration: 65 s, and temperature set point: 20 C. The identity of all specimens remained unknown to wet lab staff throughout the workflow.

### Whole Genome Sequencing Library Preparation

The paired-ends sequencing libraries were prepared using 50 µl of the fragmented DNA solution (1 µg DNA final) as input and Illumina TruSeq^®^ DNA PCR-Free Library Prep protocol HS (Illumina Inc., San Diego, CA, United States) for Whole Genome Sequencing reagent kit, following the manufacturers’ instructions. Briefly, the protocol steps were: 1) Cleanup of fragmented DNA, 2) Repair ends and selection of library size, 3) Removal of large DNA fragments, 4) Removal of small DNA fragments, 5) 3′-ends adenylation 6) Adapter ligation, and 7) Cleanup of not-ligated fragments.

### Library Quality Control

For quality control of adapter-ligated fragment sizes, libraries were diluted 1:5 with water, and 2 µl were evaluated in the automated electrophoresis analysis TapeStation System, with D1000 High Screen Tape (Agilent Technologies, Santa Clara, CA, United States). High-quality (ideal) libraries displayed only one peak around 900 bp (equivalent to ≈470 bp fragments due to the forked structures of adapter-ligated fragments) and peak molarity ≥300 p.m. Good-quality libraries had peak molarity between 200 and 300 p.m. Libraries with peak molarity ≤200 p.m. were rejected, and preparation was repeated.

### Library Pooling and Quantification

We optimized the protocol for pooling a maximum of 28 sample libraries for sequencing on each NovaSeq^®^ 6,000s4 flow cell. Briefly, each library was quantified with Qubit^®^ 4 fluorometer then normalized to 7 nM in a final volume of 11 µl. Then, we pooled the 28 libraries into a final volume of 308 µl (28 × 11 = 308 µl). Next, starting with 5 µl of the pooled solution as input, we performed two dilutions in a resuspension buffer (1:10 and 1:100, reaching the final 1:1,000 concentration). Four µl of the diluted pooled solution were used for real-time quantitative polymerase chain reaction (qPCR) on ABI 7500 real-time platform (Thermo Fisher Scientific, Waltham, MA, United States) using KAPA Library Quantification Kit (Roche, Pleasanton, CA, United States). The qPCR was performed in triplicate. In each qPCR run, six KAPA DNA standards with defined concentrations were included to produce a standard quantification curve. With the mean cycle threshold (CT) of the diluted samples, we calculated the concentration of the pooled libraries solutions via linear regression while correcting for the size-difference of the KAPA standards in relation to the adapter-ligated fragments (452 bp versus 470 bp). Each pooled library was then normalized to 3 nM final concentration.

The pooled libraries were then spiked with 1.9 µl of 2.5 nM PhiX Control v3 reagent (Illumina Inc., San Diego, CA, United States). The pooled libraries were then denatured with 77 µl of fresh 0.2 N NaOH solution, followed by homogenization by vortex (1800 RPM for 1 min), centrifugation at 280 *g* for 1 min, and incubation at room temperature for 8 min. Then, 78 µl of 400 mM Tris-HCl (pH 8.0) solution was added to the libraries pool to neutralize the NaOH. Once again, the pooled libraries solution was homogenized by vortex (1800 RPM for 1 min) and centrifuged at 280 × *g* for 1 min. The total volume (466.9 µl) of the PhiX-spiked denatured library pool solution was then transferred to NovaSeq^®^ 6,000 Reagent Kit tube and proceeded to sequencing.

### Sequencing

We performed sequencing with NovaSeq^®^ 6,000 platform using S4 flow cells with 300 cycles (150 for forward reads and 150 for reverse reads). Usually, each sequencing round was composed of 28 pooled samples as described above, using both flow cells available (total 56 samples per run). Desirable sequencing quality metrics were cluster passing filter >70% and flow cell occupation >70%.

### Bioinformatics Pipeline and Quality Control Metrics

The raw sequencing files (base call file, BCL format) were converted to FASTQ format and demultiplexed in a single step using Illumina’s *bcl2fastq* program (Illumina Inc, 2019). Illumina’s DRAGEN pipeline version 3.6.3 was used to perform all alignment and variant call (SNVs, indels, CNVs, SVs) steps. Quality control metrics are provided during each DRAGEN run.

Desirable alignment quality metrics were percentage of bases that meet Q30 score >90%, 20X minimum coverage for both whole genome and autosomes, uniformity of coverage ≥80%, median insert size >300 bp, percentage of mapped reads >98%, percentage of chimeric (supplementary) reads <5%, DNA contamination ≤2%, and percentage of mapped reads marked as duplicate <10%. Some of these thresholds were adopted from recommendations published elsewhere (Marshall et al., 2020).

The DRAGEN-generated Variant Call Format (VCF) files were validated to ensure they had the correct format, and sample- and variant-specific quality metrics were also calculated. Each sample was assessed to ensure that the percent autosomal callability was >95%, as suggested elsewhere (Marshall et al., 2020).

High-quality variants were those which passed Variant Quality Score Recalibration (VQSR) filter, had read depth (RD) ≥ 10, and genotype quality (GQ) ≥ 20 in at least 80% of the individuals in the sample; their alternative alleles were present in at least one individual with RD ≥ 10 and GQ ≥ 20, and were not located into locations with high multiallelic variation (more than four alleles, includes the non-pseudoautosomal region of X and Y chromosomes).

Functional annotation of the variants was performed with a proprietary tool, Varstation (https://varsomics.com/varstation/), developed by Hospital Israelita Albert Einstein (HIAE). The VCF files were uploaded into the service, whose workflow is based on ANNOVAR (Wang et al., 2010). The variants are then classified according to international good practices on genetic variants analyses and guidelines from the American College of Medical Genetics (ACMG) (Richards et al., 2015) and the Association for Molecular Pathology (AMP) (Li et al., 2017).

### Data Analysis

The 76 samples were separated into two different analytical groups. The first group included seven sequencing libraries corresponding to GiaB benchmark samples (NA12878, NA24385, NA24149, NA24143, NA24631, NA24694, and NA24695). The second group included the remaining 69 samples, i.e., the remaining 15 GiaB samples and the 54 in-house characterized samples.

The first group was analyzed by comparison of the VCF files generated by our Bionformatics pipeline with reference VCF files provided by GiaB (version NISTv3.3.2). Each sample had an accompanying BED file with high-confidence regions coordinates. We performed the comparison through *vcfeval* software (Real Time Genomics, Hamilton, New Zealand) (Cleary et al., 2015). Briefly, *vcfeval* quantifies the number of true positives (the variant call is present in both the reference file and our file), false positives (the variant call is absent in the reference file but present in our file), and false negatives (the variant call is present in the reference file but absent in our file). We then calculated the precision, sensitivity (recall), and F-measures with those numbers. Additionally, we stratified each file by SNVs and indels coordinates. In this step we calculated the mean of each metric mentioned above alongside 95% confidence intervals (95% CI).

The second analytical group samples were evaluated manually to assess the performance of not only SNVs detection, but also for CNVs and SVs, by comparing the pipeline output with the in-house annotation or the GiaB annotation, depending on the sample origin. Supplementary Table S1 contains a list of the samples used, quality metrics, and a summary of expected and observed variant calls.

## Results

Considering all workflows, the mean sequencing yield was 2.84 TB of data per S4 flow cell. Mean %Q30 score was 92.60% ± 1.36%, mean genomic coverage 38.96X ± 10.37X and mean uniformity was 96.31% ± 0.25%. Mean mitochondrial coverage was 7,650.97X ± 5,559.1X.

Variant calls from our WGS procedure yielded very high concordance with the reference samples. For SNVs, the mean F-measure (n = 7 reference GiaB samples) was 99.82% (95% CI = 99.44%–100.0%), whereas for indels of any length was 99.57% (95% CI = 99.29%–99.85%) (Table 1, Supplementary Table S2, Supplementary Figure S1).

**TABLE 1:** Quality metrics. Seven Genome in a Bottle Consortium gold standard samples were whole-genome sequenced, and variant call was performed with our bioinformatics pipeline. The variant call files were then compared with the gold standard files using the *vcfeval* software. Precision, Sensitivity, and F-measure are displayed.

Target	Metric	Mean	Standard deviation	95% confidence interval	
				Lower bound	Upper bound
	Precision	0.9986	0.0011	0.9965	1.0000
SNVs	Sensitivity	0.9979	0.0029	0.9922	1.0000
	F-measure	0.9982	0.0020	0.9944	1.0000
	Precision	0.9961	0.0008	0.9944	0.9977
Indels, overall	Sensitivity	0.9954	0.0021	0.9912	0.9995
	F-measure	0.9957	0.0014	0.9929	0.9985
	Precision	0.9965	0.0008	0.9949	0.9981
Indels, 1 to 5 bp	Sensitivity	0.9961	0.0019	0.9923	0.9998
	F-measure	0.9963	0.0013	0.9936	0.9989
	Precision	0.9939	0.0015	0.9910	0.9968
Indels, 6 to 15 bp	Sensitivity	0.9916	0.0028	0.9861	0.9971
	F-measure	0.9927	0.0021	0.9887	0.9968
	Precision	0.9832	0.0055	0.9725	0.9939
Indels, ≥ 16 bp	Sensitivity	0.9795	0.0082	0.9634	0.9955
	F-measure	0.9813	0.0036	0.9744	0.9883

Our procedure worked best for small indels with length between one and five base-pairs (bp) (mean F-measure = 99.63%, 95% CI = 99.36%–99.89%). Six to 15 bp indels yielded mean F-measure = 99.27%, 95% CI = 98.87%–99.68% and 16-bp or more indels yielded mean F-measure = 98.13%, 95% CI = 97.44%–98.83% (Table 1).

Our optimized WGS protocol presented excellent intra- and inter-assay reproducibility. Regarding SNVs, the intra-assay coefficient of variation (CV) of the F-measures was 0.04%, whereas the inter-assay was 0.03%. Regarding indels, the intra-assay F-measures CV was 0.16% whereas the inter-assay CV was 0.07% (Table 2).

**TABLE 2:** Reproducibility. The benchmark sample NA24385 was selected for intra-assay reproducibility evaluation, whereas NA24694 was included in all three independent workflows for inter-assay reproducibility evaluation. Coefficients of variation (CV) of quality metrics are reported.

Reproducibility	Samples	SNVs			Indels		
		Precision	Sensitivity	F-measure	Precision	Sensitivity	F-measure
	NA24385	0.9970	0.9918	0.9944	0.9969	0.9948	0.9959
	NA24385-2	0.9963	0.9914	0.9939	0.9952	0.9920	0.9936
Intra-assay	*Mean*	*0.9967*	*0.9916*	*0.9941*	*0.9961*	*0.9934*	*0.9947*
	*SD*	*0.0005*	*0.0003*	*0.0004*	*0.0012*	*0.0020*	*0.0016*
	**CV (%)**	**0.0501**	**0.0322**	**0.0411**	**0.1221**	**0.1991**	**0.1607**
	NA24694	0.9992	0.9993	0.9993	0.9968	0.9980	0.9974
	NA24694-2	0.9994	0.9993	0.9993	0.9974	0.9986	0.9980
Inter-assay	NA24694-3	0.9988	0.9989	0.9988	0.9963	0.9968	0.9966
	*Mean*	*0.9991*	*0.9991*	*0.9991*	*0.9968*	*0.9978*	*0.9973*
	*SD*	*0.0003*	*0.0002*	*0.0003*	*0.0005*	*0.0009*	*0.0007*
	**CV (%)**	**0.0312**	**0.0235**	**0.0271**	**0.0551**	**0.0909**	**0.0726**

SD, standard deviation; CV, coefficient of variation.

The pathogenic/likely pathogenic variant profile of the 54 in-house characterized samples included: 12 SNVs (eight missense, two nonsense, one splicing acceptor, and another splicing donor), 65 large deletions (lengths ranging between 538 bp–53, 247, 491 bp), including 29 loss of heterozygosity (LOH) regions identified by SNP array (lengths ranging between 812,863 bp and 72, 740, 279 bp); 22 large duplications (ranging between 6,147 bp and 95, 325, 642 bp), three events of trisomy (chromosomes 13, 15 or 21), one insertion, four inversions, ten translocations, two Robertsonian translocations and a single occurrence of uniparental disomy, totaling 120 events.

All SNVs were correctly detected by our variant call procedure (F-measure = 100.0%). The detection of the single event of uniparental disomy failed (Table 3). The CNV and SV events detection performances were overall good (F-measures 96.6% and 90.3%, respectively).

**TABLE 3:** Quality metrics of the variant call procedure were performed on 69 samples, including 54 in-house characterized samples by other methodologies. The seven GiaB gold-standard samples are not considered here; see Table 1. Also, see Supplementary Table 1 for a breakdown of expected and observed variant calls (analysis group 2 rows).

Variant	True positives (TP)	False negatives (FN)	Precision	Sensitivity	F-measure
SNVs					
Missense	8	0	1.0000	1.0000	1.0000
Nonsense	2	0	1.0000	1.0000	1.0000
Splicing acceptor	1	0	1.0000	1.0000	1.0000
Splicing donor	1	0	1.0000	1.0000	1.0000
Overall	***12***	***0***	***1.0000***	***1.0000***	***1.0000***
CNVs					
Deletions	60	5	1.0000	0.9231	0.9600
Duplications	22	0	1.0000	1.0000	1.0000
Trisomy 13	1	0	1.0000	1.0000	1.0000
Trisomy 15	0	1	Not calculated	0.0000	0.0000
Trisomy 21	1	0	1.0000	1.0000	1.0000
Overall	***84***	***6***	***1.0000***	***0.9333***	***0.9655***
SVs					
Insertions	1	0	1.0000	1.0000	1.0000
Inversions	4	0	1.0000	1.0000	1.0000
Robertsonian translocations	0	2	Not calculated	0.0000	0.0000
Translocations	9	1	1.0000	0.9000	0.9474
Overall	***14***	***3***	***1.0000***	***0.8235***	***0.9032***
Uniparental disomy (UPD)	0	1	Not calculated	0.0000	0.0000
SNVs + CNVs + SVs + UPD	***110***	***10***	***1.0000***	***0.9167***	***0.9565***

Currently, we have sequenced over 2,000 among 3,000 enrolled patients with rare diseases or hereditary cancer syndromes with our optimized WGS protocol. Sequencing and alignment metrics are available for about 1,200 samples and have been consistently high-quality, compatible with clinical diagnostic workflow (Supplementary Table S3). For example, cross-individual contamination is virtually non-existent (men 0.008% ± 0.11), the mean uniformity of coverage is 96.4% ± 0.26%, the median genome coverage is 36.5X, the mean percentage of bases with quality score Q30 or more is 91.3% ± 3.6% and mean genome callability is 96.3% ± 1.2%. Of those, over 300 patients have received a diagnostic report, with approximately 37% presenting a definitive molecular diagnosis, with the detection of a pathogenic/likely pathogenic variant compatible with the patient’s phenotype.

## Discussion

The diagnosis of patients with rare disorders is currently a lengthy process, taking four or more years on average. Early adoption of WGS could be beneficial, shortening the diagnostic odyssey (Wu et al., 2020; Rehm, 2022). A recent meta-analysis of 37 studies involving children with genetic diseases showed that WGS testing had higher clinical utility (ACMG Board of Directors, 2015) than chromosomal microarray. An accompanying meta-regression showed that the odds of diagnosis through WGS increased by 16% each year, possibly due to methodological improvements (the meta-analysis included WGS studies published between 2015 and 2017) (Clark et al., 2018). Other studies reported (Costain et al., 2020)the clinical utility of rapid WGS for children undergoing intensive care (Sanford et al., 2019).

A recent application of WGS to rare diseases diagnosis in a national context (the United Kingdom 100 K Genomes Project) revealed a remarkable benefit to routine healthcare (Turro et al., 2020). A meta-analysis of psychological outcomes suggested no harm following WGS result disclosure and even an overall trend for a decrease in anxiety (Robinson et al., 2019). Thus, it is becoming increasingly clearer that genomic medicine can revolutionize the healthcare of an individual with a rare disease or cancer by offering prompt and accurate diagnosis, risk stratification based upon genotype, and the ability for personalized treatments.

Brazil is the only country with a population larger than 100 million people, which has a public, universal, and free of charge health care system (Castro et al., 2019). Thus, provisioning a cost-effective genomic testing strategy within a national healthcare service to deliver equity of access is challenging (Berg et al., 2017), with a system of this magnitude. To further our progress in the area, we are performing a pilot project for the use of WGS for the diagnosis of rare diseases (The Rare Genomes Project - www.genomasraros.com) in Brazil, which will sequence over 9,000 individuals until the end of 2023.

To this end, we developed and validated a comprehensive WGS workflow with an optimized laboratory turnaround time coupled with a cutting-edge bioinformatics pipeline for variant calling, functional annotation, and classification. Our procedure was performed following important benchmarking guidelines (Krusche et al., 2018; Koboldt, 2020) and yielded excellent performance. One critical step for robust validation is careful sample selection. Using a set composed of reference benchmark samples, which have millions of completely validated variants of different types, and in-house characterized or purchased samples for more complex variants such as structural, mitochondrial, and LOH events is of paramount importance. In addition, the validation of detection of hard-to-detect variant types, such as repeat expansions, variants in genes with pseudogenes or homologous genes, and low-level mosaicism, requires even further steps, including additional samples, possibly on a gene-by-gene basis (Marshall et al., 2020).

Assessing and interpreting variants is challenging, and we acknowledge some limitations of our protocol. For example, we did not evaluate repeat expansion variants, tandem duplications, mitochondrial genome heteroplasmy, mosaicism, and processed pseudogene insertions. Moreover, only CNVs over than 500 bp were detected using our pipeline. Therefore, the detection sensitivity of CNVs with less than 538 bp may differ from the one reported here. We plan soon to validate the detection of some of these variant types to improve the test robustness, sensitivity, specificity, and detection limits. Moreover, we are currently developing ancestry analysis pipelines to describe and quantify ancestry in the Brazilian Rare Genomes Project participants. Brazilian populations. Population substructure and genetic ancestry are fundamental issues to consider when assessing rare diseases. Brazilian populations are admixed, with each individual having a substantial genetic contribution from European, African, and Amerindian ancestral populations. In general, European genomic contribution is most represented, followed by the African and then the Amerindian contribution (Pena et al., 2009).

## Conclusion

Large-scale WGS projects are important initiatives to expand the population’s access to these robust genomic technologies. The validation of our WGS workflow is the first step for this achievement. It has the potential to reduce the time until diagnosis of patients with rare diseases, improving the affected individuals and their family’s quality of life. Also, considering the high diversity of our population, The Rare Genomes Project is fundamental for creating a disease-related variants database, contributing with the future of precision medicine in this country.

## Data Availability

The original contributions presented in the study are included in the article/Supplementary Material, further inquiries can be directed to the corresponding author.

## Supplementary Material

The Supplementary Material for this article can be found online at: https://www.frontiersin.org/articles/10.3389/fmolb.2022.821582/full#supplementary-material

Supplementary Table S1 List of samples in the validation dataset. Analysis group 1 (7 reference samples from Genome in a Bottle Consortium) was evaluated with vcfeval software. Analysis group 2 (69 samples) was evaluated through manual curation. For analysis group 2, variant types expected and observed variant calls are provided. The column Interpretation indicates if the calls match (TP = true positive) or not (FN = false negative). Sequencing and alignment quality metrics are provided.

Supplementary Table S2 Quality metrics per reference sample (n = 7 reference samples from Genome in a Bottle Consortium, with one being in duplicate and the other being in triplicate). Number of false negatives (FN), false positives (FP), and true positives (TP) are displayed.

Supplementary Table S3 Sequencing and alignment metrics for the first samples sequenced by the Brazilian Rare Genomes Project (n = 1,206). Metrics have been consistently of high quality, compatible with clinical diagnostic routine.

Supplementary Figure S1 Circular plot displaying variant calls in the seven gold-standard GiaB samples. They are ordered according to chromosomes (circular sectors) and genomic position. Each library corresponds to one circumference of said sectors. Yellow points/top third of the sector: true positive calls, red points/middle third of the sector: false-positive calls, blue points/bottom third of the sector: false-negative calls. Left: result for SNVs, right: result for indels.

## References

[B1] ACMG Board of Directors (2015). Clinical Utility of Genetic and Genomic Services: a Position Statement of the American College of Medical Genetics and Genomics. Genet. Med. 17 (6), 505–507. 10.1038/gim.2015.41 25764213

[B2] Aziz N. Zhao Q. Bry L. Driscoll D. K. Funke B. Gibson J. S. (2015). College of American Pathologists' Laboratory Standards for Next-Generation Sequencing Clinical Tests. Arch. Pathol. Lab. Med. 139 (4), 481–493. 10.5858/arpa.2014-0250-cp 25152313

[B3] Barra G. B. Júnior N. G. Filho J. B. O. (2018). Lista de Orientação em Diagnóstico Molecular. Segunda versão. Rio de Janeiro, Brazil: Sociedade Brasileira de Patologia Clínica/Medicina Laboratorial (SBPC/ML).

[B4] Belkadi A. Bolze A. Itan Y. Cobat A. Vincent Q. B. Antipenko A. (2015). Whole-genome Sequencing Is More Powerful Than Whole-Exome Sequencing for Detecting Exome Variants. Proc. Natl. Acad. Sci. 112 (17), 5473–5478. 10.1073/pnas.1418631112 25827230 PMC4418901

[B5] Berg J. S. Agrawal P. B. Bailey D. B. Jr. Beggs A. H. Brenner S. E. Brower A. M. (2017). Newborn Sequencing in Genomic Medicine and Public Health. Pediatrics 139 (2), e20162252. 10.1542/peds.2016-2252 28096516 PMC5260149

[B6] Bick D. Jones M. Taylor S. L. Taft R. J. Belmont J. (2019). Case for Genome Sequencing in Infants and Children with Rare, Undiagnosed or Genetic Diseases. J. Med. Genet. 56 (12), 783–791. 10.1136/jmedgenet-2019-106111 31023718 PMC6929710

[B8] Castro M. C. Massuda A. Almeida G. Menezes-Filho N. A. Andrade M. V. de Souza Noronha K. V. M. (2019). Brazil's Unified Health System: the First 30 Years and Prospects for the Future. Lancet. 394 (10195), 345–356. 10.1016/s0140-6736(19)31243-7 31303318

[B10] Clark M. M. Stark Z. Farnaes L. Tan T. Y. White S. M. Dimmock D. (2018). Meta-analysis of the Diagnostic and Clinical Utility of Genome and Exome Sequencing and Chromosomal Microarray in Children with Suspected Genetic Diseases. npj Genomic Med. 3 (1), 16. 10.1038/s41525-018-0053-8 PMC6037748 30002876

[B11] Cleary J. G. Braithwaite R. Gaastra K. Hilbush B. S. Inglis S. Irvine S. A. (2015). Comparing Variant Call Files for Performance Benchmarking of Next-Generation Sequencing Variant Calling Pipelines. bioRxiv., 023754. 10.1101/023754

[B12] Costain G. Walker S. Marano M. Veenma D. Snell M. Curtis M. (2020). Genome Sequencing as a Diagnostic Test in Children With Unexplained Medical Complexity. JAMA Netw. Open. 3 (9), e2018109. 10.1001/jamanetworkopen.2020.18109 32960281 PMC7509619

[B13] Ferreira C. R. (2019). The burden of Rare Diseases. Am. J. Med. Genet. A. 179 (6), 885–892. 10.1002/ajmg.a.61124 30883013

[B14] Howell K. B. Eggers S. Dalziel K. Riseley J. Mandelstam S. Myers C. T. (2018). A Population-Based Cost-Effectiveness Study of Early Genetic Testing in Severe Epilepsies of Infancy. Epilepsia. 59 (6), 1177–1187. 10.1111/epi.14087 29750358 PMC5990455

[B15] Illumina Inc (2019). bcl2fastq2 Conversion Software v2.20. Retrieved March 17, 2021. Available at: https://support.illumina.com/content/dam/illumina-support/documents/documentation/software_documentation/bcl2fastq/bcl2fastq2-v2-20-software-guide-15051736-03.pdf .

[B16] Koboldt D. C. (2020). Best Practices for Variant Calling in Clinical Sequencing. Genome Med. 12 (1), 91. 10.1186/s13073-020-00791-w 33106175 PMC7586657

[B17] Krusche P. Trigg L. Boutros P. C. Mason C. E. La Vega F. M. D. Moore B. L. (2018). Best Practices for Benchmarking Germline Small Variant Calls in Human Genomes. bioRxiv, 270157. 10.1101/270157 PMC6699627 30858580

[B18] Lander E. S. Linton L. M. Birren B. Nusbaum C. Zody M. C. Baldwin J. (2001). Initial Sequencing and Analysis of the Human Genome. Nature. 409 (6822), 860–921. 10.1038/35057062 11237011

[B19] Li M. M. Datto M. Duncavage E. J. Kulkarni S. Lindeman N. I. Roy S. (2017). Standards and Guidelines for the Interpretation and Reporting of Sequence Variants in Cancer: A Joint Consensus Recommendation of the Association for Molecular Pathology, American Society of Clinical Oncology, and College of American Pathologists. J. Mol. Diagn. 19 (1), 4–23. 10.1016/j.jmoldx.2016.10.002 27993330 PMC5707196

[B20] Lionel A. C. Costain G. Monfared N. Walker S. Reuter M. S. Hosseini S. M. (2018). Improved Diagnostic Yield Compared with Targeted Gene Sequencing Panels Suggests a Role for Whole-Genome Sequencing as a First-Tier Genetic Test. Genet. Med. 20 (4), 435–443. 10.1038/gim.2017.119 28771251 PMC5895460

[B21] Marshall C. R. Chowdhury S. Taft R. J. Lebo M. S. Buchan J. G. Harrison S. M. (2020). Best Practices for the Analytical Validation of Clinical Whole-Genome Sequencing Intended for the Diagnosis of Germline Disease. npj Genomic Med. 5 (1), 47. 10.1038/s41525-020-00154-9 PMC7585436 33110627

[B22] Nagarajan N. Pop M. (2013). Sequence Assembly Demystified. Nat. Rev. Genet. 14 (3), 157–167. 10.1038/nrg3367 23358380

[B23] Pena S. D. Bastos-Rodrigues L. Pimenta J. R. Bydlowski S. P. (2009). DNA Tests Probe the Genomic Ancestry of Brazilians. Braz. J. Med. Biol. Res. 42 (10), 870–876. 10.1590/s0100-879x2009005000026 19738982

[B24] Rehm H. L. (2022). Time to Make Rare Disease Diagnosis Accessible to All. Nat. Med. 28 (2), 241–242. 10.1038/s41591-021-01657-3 35132266 PMC8866216

[B25] Richards S. Aziz N. Bale S. Bick D. Das S. Gastier-Foster J. (2015). Standards and Guidelines for the Interpretation of Sequence Variants: a Joint Consensus Recommendation of the American College of Medical Genetics and Genomics and the Association for Molecular Pathology. Genet. Med. 17 (5), 405–424. 10.1038/gim.2015.30 25741868 PMC4544753

[B26] Robinson J. O. Wynn J. Biesecker B. Biesecker L. G. Bernhardt B. Brothers K. B. (2019). Psychological Outcomes Related to Exome and Genome Sequencing Result Disclosure: a Meta-Analysis of Seven Clinical Sequencing Exploratory Research (CSER) Consortium Studies. Genet. Med. 21 (12), 2781–2790. 10.1038/s41436-019-0565-3 31189963 PMC7260995

[B27] Sanford E. F. Clark M. M. Farnaes L. Williams M. R. Perry J. C. Ingulli E. G. (2019). Rapid Whole Genome Sequencing Has Clinical Utility in Children in the PICU. Pediatr. Crit. Care Med. 20 (11), 1007–1020. 10.1097/pcc.0000000000002056 31246743 PMC6832787

[B29] The 1000 Genomes Project Consortium (2015). A Global Reference for Human Genetic Variation. Nature 526 (7571), 68–74. 10.1038/nature15393 26432245 PMC4750478

[B30] Turro E. Astle W. J. Megy K. Gräf S. Greene D. Shamardina O. (2020). Whole-genome Sequencing of Patients with Rare Diseases in a National Health System. Nature 583 (7814), 96–102. 10.1038/s41586-020-2434-2 32581362 PMC7610553

[B31] Venter J. C. Adams M. D. Myers E. W. Li P. W. Mural R. J. Sutton G. G. (2001). The Sequence of the Human Genome. Science 291 (5507), 1304–1351. 10.1126/science.1058040 11181995

[B32] Vissers L. van Nimwegen K. J. M. Schieving J. H. Kamsteeg E. J. Kleefstra T. Yntema H. G. (2017). A Clinical Utility Study of Exome Sequencing versus Conventional Genetic Testing in Pediatric Neurology. Genet. Med. 19 (9), 1055–1063. 10.1038/gim.2017.1 28333917 PMC5589982

[B33] Wang K. Li M. Hakonarson H. (2010). ANNOVAR: Functional Annotation of Genetic Variants from High-Throughput Sequencing Data. Nucleic Acids Res. 38 (16), e164. 10.1093/nar/gkq603 20601685 PMC2938201

[B34] Wetterstrand K. A. (2020). DNA Sequencing Costs: Data from the NHGRI Genome Sequencing Program (GSP). Retrieved March 17, 2021. Available at: https://www.genome.gov/about-genomics/fact-sheets/DNA-Sequencing-Costs-Data .

[B35] Wu A. C. McMahon P. Lu C. (2020). Ending the Diagnostic Odyssey-Is Whole-Genome Sequencing the Answer? JAMA Pediatr. 174 (9), 821–822. 10.1001/jamapediatrics.2020.1522 32597967 PMC7928067

