# Supplementary figures and images for: The Brazilian Rare Genomes Project: Validation of Whole Genome Sequencing for Rare Diseases Diagnosis
Source: Front Mol Biosci. 2022 May 2;9:821582. doi: 10.3389/fmolb.2022.821582 (PMC9108541; doi:10.3389/fmolb.2022.821582)

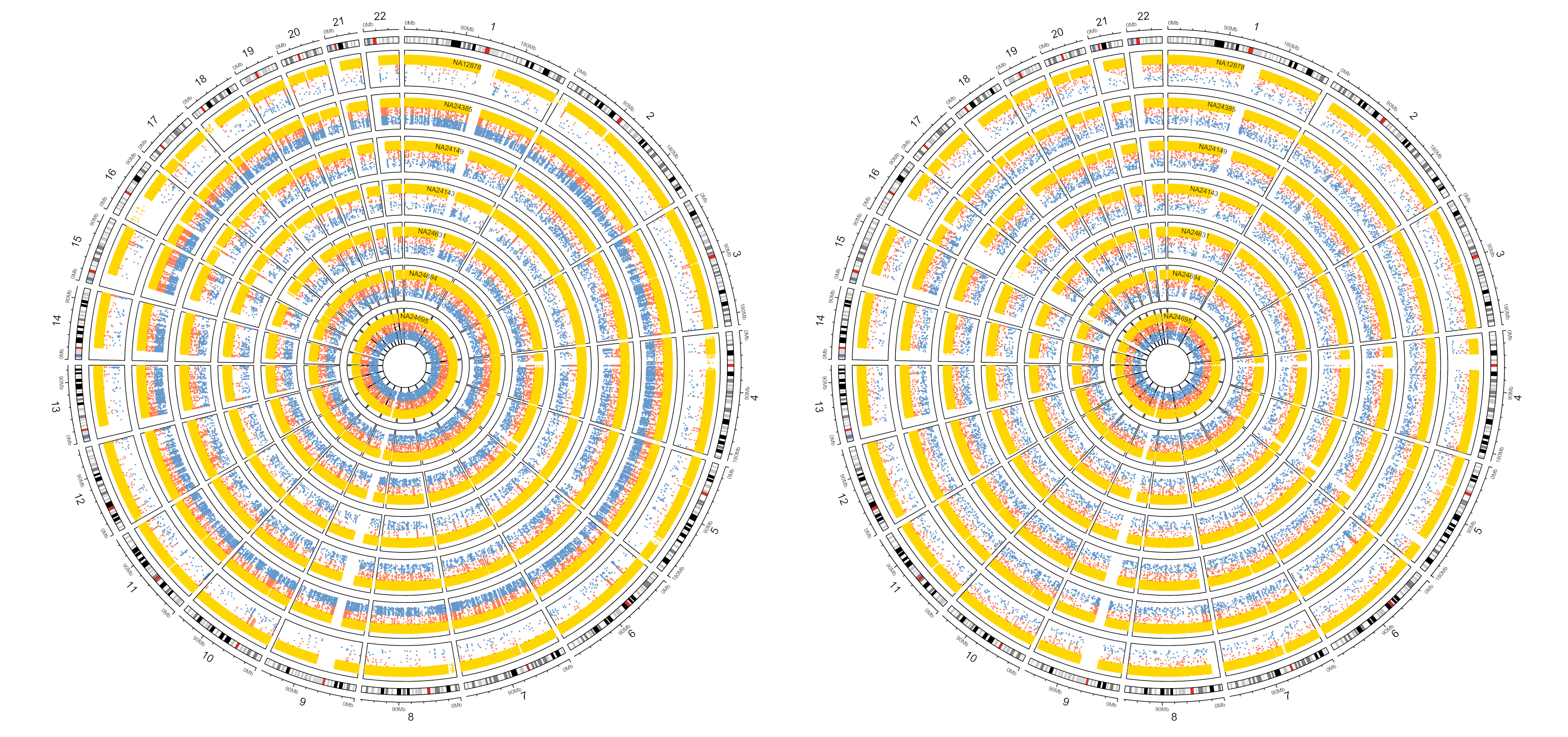

Supplement: Supplementary file 3 [file Image1.TIF]
